# “Maybe a little bit of guilt isn’t so bad for the overall health of an individual”: a mixed-methods exploration of young adults’ experiences with calorie labelling

**DOI:** 10.1186/s12889-022-13364-w

**Published:** 2022-05-10

**Authors:** Amanda Raffoul, Brooke Gibbons, Karla Boluk, Elena Neiterman, David Hammond, Sharon I. Kirkpatrick

**Affiliations:** 1 School of Public Health Sciences, University of Waterloo, 200 University Ave W, Waterloo, ON N2L 3G1 Canada; 2 Present address: Division of Adolescent/Young Adult Medicine, Department of Pediatrics, Boston Children’s Hospital, 333 Longwood Avenue, Boston, MA 02115 USA; 3 Department of Recreation and Leisure Studies, University of Waterloo, 200 University Ave W, Waterloo, ON N2L 3G1 Canada

**Keywords:** Calorie labelling, Young adults, Mixed-methods, Disordered eating, Food policy

## Abstract

**Background:**

Menu labelling, and more specifically calorie labelling, has been posited as an intervention to improve nutrition literacy and the healthfulness of consumers’ food purchases. However, there is some concern calorie labelling may unintentionally trigger or exacerbate disordered eating among vulnerable persons. The purpose of this research was to explore young adults’ experiences with labelling, with a focus on its implications for their relationships with food.

**Methods:**

Individual semi-structured interviews were conducted with participants from a campus-based menu labelling study. Interview data were inductively coded using thematic analysis and supported by survey data assessing disordered eating, body esteem, and related constructs.

**Results:**

The sample consisted of 13 participants (10 women, 3 men), most of whom perceived themselves as “about the right weight” (62%). Four key themes included: (1) participants’ support of and skepticism about labelling interventions, (2) the identification of knowledge and autonomy as mechanisms of labelling interventions, (3) the role of the individual’s and others’ relationships with food in experiences with labelling, and (4) disordered eating and dieting as lenses that shape experiences with interventions. Participants’ perceptions of and experiences with calorie labels were shaped by gender, body esteem, and disordered eating risk.

**Conclusions:**

The results provide insight into the complexity of young adults’ interactions with labelling interventions and context for future research exploring the unintended consequences of public health nutrition interventions.

**Supplementary Information:**

The online version contains supplementary material available at 10.1186/s12889-022-13364-w.

## Background

Rising weights and body sizes over the past few decades have resulted in increased attention to reducing and preventing weight gain among individuals [1]. The global ‘war on obesity’ has predominantly focused on weight loss and addressing the physiological risks associated with higher weights [2] while neglecting possible psychosocial consequences, such as internalized weight bias [3]. Weight-centric approaches may promote *healthism*, which places responsibility for health on the individual, such that illness or poor health represent a moral failing of the individual rather than the state [4]. For example, critical reviews have illustrated the framing of higher weights within public health as an ‘individual problem’ [5, 6], with lesser consideration of the role of social determinants of health. Weight-related public health interventions often also emphasize individual *agency* versus *structure* in their promotion of ‘healthy weights’ [7].

One increasingly popular policy approach to reduce weights is menu and front-of-package labelling [8]. Such labels may provide an overall representation of the ‘healthfulness’ of a food (e.g., traffic light labelling) or convey numeric information about particular dietary components (e.g., amounts of sodium, sugars, or saturated fat), but increasingly, they focus on calories. In tandem with regulations and proposals related to labelling interventions in numerous countries [9], there are concerns about their potential to elicit unintended consequences for disordered eating and eating disorders [10]. Disordered eating is characterized by attitudes and behaviours, such as severe caloric restriction or self-induced vomiting, that are intended to modify weight and are harmful to health [11]. Disordered eating affects up to 30% of young adults [12, 13], is most prevalent among women [14, 15] and individuals with higher weights [14, 16], and can subsequently increase risk of eating disorders, psychiatric illnesses characterized by significant impairment to wellbeing.

Calorie labels may oversimplify the nutritional and social values of food [17, 18] (though generally, the caloric content of foods is correlated with its overall healthfulness [19]) and reinforce behaviours associated with disordered eating, such as calorie counting [20]. Individuals trying to modify their weight actively seek out nutrition information [21, 22] and those engaged in disordered eating appear more likely to use labels than those who are not [23, 24]. In an online retail simulation that exposed individuals to hypothetical calorie labels, individuals with eating disorders expressed support for menu labels, and those with anorexia or bulimia nervosa stated they would order items with fewer calories and those with binge eating disorder opted for items with more calories compared to individuals without eating disorders [25]. However, a pre-post campus-based experimental study found calorie labels did not worsen eating disturbance among undergraduate student women after 1 month of implementation [26].

One mixed-methods study found some young adults recognize that labelling may elicit negative consequences for individuals with disordered eating [27], but quantitative evidence suggests many young adults report support for such policies [28] and do not perceive labels as harsh [29]. This seemingly contradictory policy support may be reflective of a desire for transparency at the point of food purchase, along with societal norms that emphasize individual responsibility in achieving and maintaining healthy eating and weights [30]. There is a paucity of research exploring how individuals experience labelling, particularly in relation to disordered eating risk, but mixed-methods research has been suggested as a means of exploring the unintended consequences of such interventions [31]. Accordingly, we conducted a mixed-methods study to explore how young adults feel about, perceive, and experience weight-related population-level interventions, with a focus on calorie labelling. Furthermore, we sought to explore how young adults’ personal characteristics (gender, body esteem, and disordered eating risk) shape their attitudes, perceptions, and experiences.

## Methods

**Fig. 1:**
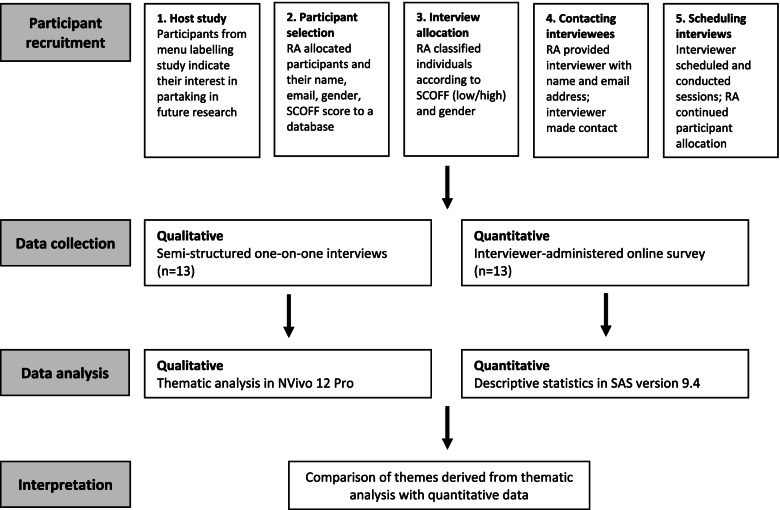
Recruitment and convergent mixed-methods study design

This study used a convergent mixed-methods design (Fig. 1) [32]. Qualitative data were yielded by semi-structured, one-on-one interviews, and quantitative data were collected using a survey consisting of socio-demographic and food- and body-related measures. The study was reviewed by and received clearance from the University of Waterloo Office of Research Ethics (ORE #40501). All methods were carried out in accordance with the relevant guidelines and regulations set by the University of Waterloo.

### Participants and recruitment

Participants were recruited from a larger quasi-experimental controlled study (*n* = 1623) conducted at the University of Waterloo in 2019. This study used a pre- and post-intervention design to evaluate young adults’ noticing of, use, and perceptions of traffic light and numeric calorie labels, as well as the impact of labels on food and beverage purchasing. Calorie labelling was introduced at two residence cafeterias, which were randomized to numeric or traffic light labels, while a third residence cafeteria received no labels. The traffic light labels presented caloric information within a green circle for low-calorie, amber circle for middle-calorie, or red circle for high-calorie food and beverage items [33]. Calorie labelling was also in place in chains with more than 20 outlets in the province in which the study was conducted [34], and it was assumed participants had some baseline exposure to labels.

Participants in cafeterias were approached by research staff as they left the venue, and those who consented to participate in the study were invited to complete exit surveys on iPads and received a $5 CAD remuneration for their participation [35]. Those who expressed interest in related research (*n* = 343) represented the sampling frame for the present study (Fig. 1). Purposive sampling via maximum variation was used [36], with the aim of achieving variation across disordered eating status, as well as gender. A research assistant categorized participants into one of six groups based on gender identity (man or woman or trans/nonbinary) and disordered eating risk (low/high). Disordered eating risk was assessed using SCOFF, a 5-item measure used among non-clinical populations [37] whereby endorsement of at least 2 items indicates high disordered eating risk. The research assistant provided potential participants’ contact information to the interviewer, who contacted those eligible via email requesting participation in an interview and survey aiming to explore young adults’ feelings, perceptions, and experiences with population-level nutrition policies. Participant recruitment was iterative, with small groups of participants emailed over a period of 2 weeks, to achieve a diverse sample by gender and disordered eating status. A total 79 participants were invited to participate via email; no participants declined, but most (*n* = 66) did not respond to the initial or follow-up email. Overall, thirteen participants were interviewed in March and April 2019, at which point data collection was ceased. Participants were provided with $15 CAD cash remuneration.

### Data collection

#### Semi-structured interviews

After providing written informed consent, each participant took part in an in-person semi-structured interview, following an interview guide that explored participants’ thoughts on policy and food labelling, including potential implications on their own and other students’ relationships with food (see Additional file 1). Interviews were approximately an hour in length and were recorded and transcribed verbatim.

#### Surveys

Following the interview, participants completed a short survey, hosted on a Qualtrics server, that queried age, gender identity [38], and race/ethnicity [39]. Weight perception was measured by asking participants whether they consider themselves ‘overweight’, ‘underweight’, ‘just about right’, ‘don’t know’, or ‘refuse to answer’ [39]. Body esteem was assessed using the Body Esteem Scale for Adolescents and Adults (BESAA), a 23-item trait measure that uses a five-point Likert scale to indicate frequency of agreement from 0 (never) to four (always). BESAA consists of three subscales (appearance, weight, and attribution) with high internal consistency (Cronbach’s α = 0.92, 0.81, 0.94) and measures body-related self-evaluation among adolescents and young adults across genders [40]; higher scores reflect greater body esteem. Finally, the 26-item Eating Attitudes Test (EAT-26) was used to measure disordered eating attitudes and behaviours. EAT-26 is a shortened (26 item) version of the 40-item EAT but more detailed and specific than brief measures such as SCOFF [41]. The EAT-26 uses a six-point Likert scale to indicate frequency of agreement from one (never) to six (always) and consists of three subscales (dieting, bulimia, and food preoccupation/control) with high sensitivity; higher scores indicate greater eating pathology. A score of 20 or higher is indicative of severe eating pathology, although a score below 20 does not exclude the possibility of disordered eating [41].

### Analyses

Aligning with the convergent mixed-methods design [32], quantitative and qualitative data were analysed separately and integrated during the final analytic stage (Fig. 1). Quantitative data were analyzed using SAS, version 9.4 (SAS Institute, Cary, North Carolina). Descriptive statistics were derived to describe the sample characteristics, including mean BESAA and EAT-26 scores.

Interview transcripts were analyzed using NVivo 12 Pro (QSR International Pty Ltd., Doncaster, Victoria, Australia). Transcripts were inductively coded and analyzed by two independent researchers using a thematic analysis framework [42]. Prior to selective coding, the quantitative and qualitative data were combined in NVivo 12 Pro, allowing for cross-comparison of codes and themes across the whole sample and then by variables of interest, including gender, body esteem, EAT-26 scores, and the four EAT-26 behavioural items. The primary researcher explored cross-comparisons independently and all final themes were reviewed by the research team.

## Results

Participant characteristics (*n* = 13) are summarized in Table 1. An overview of the themes and subthemes are displayed in Table 2 and detailed in the following sections.

**Table 1:** Participant demographic characteristics

Characteristic	% (n)
**Age (mean (SD))**	18.8 (1.3)
**Gender**	
Man	23.1 (3)
Woman	76.9 (10)
**Race/ethnicity**	
Caribbean	23.1 (3)
East Asian (e.g., Chinese, Japanese, Korean)	15.4 (2)
South Asian (e.g., East Indian, Pakistani, Sri Lankan)	30.8 (4)
Southeast Asian (e.g., Cambodian, Indonesian, Laotian, Vietnamese)	7.7 (1)
White	23.1 (3)
**Weight perception**	
“Underweight”	15.4 (2)
“About the right weight”	61.5 (8)
“Overweight”	23.1 (3)
**BESAA score (mean (SD))**	51.0 (13.5)
Participants below average	46.2 (6)
Participants at or above average	53.8 (7)
**EAT-26 score (mean (SD))**	7.7 (5.5)
Participants below average	61.5 (8)
Participants at or above average	38.5 (5)

*BESAA* Body Esteem Scale for Adolescents and Adults, a 23-item measure that measures body-related self-evaluation among young adults across genders; higher scores reflect greater body esteem, with scores ranging from 0 to 92 [39]. *EAT-26* Eating Attitudes Test-26, higher scores indicate greater eating pathology, with scores ranging from 0 to 26 [40]

**Table 2:** Overview of major themes and subthemes

Major theme	Corresponding subthemes
Support of & skepticism about labelling interventions	Policy support for menu labelling Skepticism towards food policy or labels
Knowledge & autonomy as mechanisms of population-level interventions	Awareness, education, and information - Calorie awareness - Food allergies or intolerances Noticing and use of labels - Counting calories & doing math - Colour associations of labels Obesity and health consequences of poor diets
Role of the relationship with food in experiences with labelling	Personal connections with food Food & relationships with others/Societal pressures surrounding food Short- and long-term influences of labels Negative impact of labels on relationship with food
Disordered eating and dieting as a lens in experiences with interventions	Overindulging and/or bingeing Regret and shame associated with food Restrictive food and/or dieting behaviour Speaking for a friend

### Support of & skepticism about labelling interventions

Most participants voiced support for the intention of labelling interventions to improve population health, but also skepticism related to the capacity of labels to change their own and others’ food-related attitudes and behaviours. *Policy support* for labelling and related policies (e.g., dietary guidance) was exhibited by eleven participants, who detailed the perceived benefits of such policies for themselves and/or others. Jen, a 23-year old international student from China, explained:

*Those who want to know, and before they never had the resources or there’s no way for them to know the exact calorie content in one specific things, now they—they're being provided this opportunity … which hopefully can change their purchasing decision and help them make more healthier choices.*

Participants described how having some information about the nutritional content of their food was better than none, and that the clarity and perceived usefulness of information on menu labels through displays of calories and/or traffic lights was high.

On the other hand, twelve participants were *skeptical about food policies and/or labels*. While some participants liked and/or used labels, most also identified potential limitations in terms of their effectiveness, particularly among young adult populations. For example, Cassie, an 18-year old White woman, noted:

*We know that we’re supposed to eat healthy and exercise and do all those things, but we’re still not doing it. Changing policy isn’t necessarily going to change the way that people think about food and how they consume it.*

Several participants described considerations influencing food decisions beyond personal choice and the use of labels, including financial resources and access to cooking facilities, the convenience of fast foods, and time to buy and prepare healthy foods.

### Knowledge & autonomy as mechanisms of labelling interventions

All participants identified *awareness, education, and information* about the caloric content of foods and beverages as the avenue through which labels may lead individuals to make healthier decisions. For example, Maya, a 19-year-old South Asian woman, stated: *‘I think it’s more about being aware, rather than making a change. They want to let people know.’* Some participants identified other values of labels, such as avoiding allergens.

In describing the usefulness of labels to inform purchases, eight participants exhibited *calorie awareness*, detailing the meaning of calories and the calorie content of certain foods. Some participants highlighted the shortcomings of a focus on calories, including Cassie: *‘Some healthy foods like nuts, like healthy fats and oils, those are great for you but they might be higher in calories than something that’s not necessarily good for you.’* The five participants who did not exhibit calorie awareness in their interviews were international students. There were no differences in *calorie awareness* by disordered eating risk, though participants who did not speak about calories did have slightly higher body esteem scores; participants who exhibited calorie awareness had an average EAT-26 score of 7.5 (SD = 3.9) and an average BESAA score of 48.3 (SD = 9.7), while participants who did not speak about calories had mean scores of 8 (SD = 8.0) and 55.4 (SD = 18.5), respectively.

All participants drew attention to the *noticing and use of labels*. Rahul, an 18-year old South Asian male, detailed that although he does not care about caloric content, he still notices labels when ordering food, ‘*not … intentionally, but … because it’s bright and it’s saying something.’* Participants with disordered eating scores above the sample average spent more time detailing their experience in interacting with a calorie label and provided longer responses to the questions about the labels than participants whose scores were within one point of the average and/or below it.

In discussions about traffic light labels, eight participants, who mostly had average body esteem and elevated disordered eating scores, described the *value assigned to the colours of labels* in traffic light labels and how different colours aligned with knowledge about calories and their associations with the healthfulness of foods. For example, Emily, an 18-year-old woman, detailed *‘the associations that people have, like cultural associations with red, yellow, and green is like: Red is bad. Yellow, ehh. And green is good.’* Relatedly, there was some concern regarding the lack of clarity about what the colours mean. For example, one participant said she did not know how it was determined what was a high- versus medium-calorie item, but suggested the labelling of an item with red means ‘*it must be bad*’.

Pertaining to *autonomy as a mechanism* of labels, whereby individual choice and action was considered the pathway through which labels worked, nine participants detailed *counting calories*, often associated with ‘doing the math’*,* as a component of ordering foods when calorie labels were present. Although several described counting calories as a tool others could employ, Monica, a 19-year-old East Asian woman with a previous eating disorder, described how doing the math influenced her use of labels:

*I remember like, even calculating for one of the drinks because, um, I don’t know if you’ve had bubble tea yourself, but you can change like the ice levels and the sugar levels, and I remember like, trying to meticulously calculate what—what it was like.*

Of the nine participants who discussed counting, four indicated counting calories themselves, while the remaining five assumed counting benefits others people who are doing it. The four participants who did not mention counting calories or ‘doing the math’ had low disordered eating risk (M = 4.8, SD = 3.6) and above-average body esteem scores (M = 52.2, SD = 17.1) compared to those who did mention counting calories (EAT-26 M = 9.0, SD = 5.9; BESAA M = 42.9, SD = 9.5).

Nine participants also described the role labelling policies play in preventing and/or reducing *obesity and related health consequences*; they were nearly split on whether the onus of responsibility for preventing weight gain and chronic disease was the government’s (i.e., by changing environmental factors) or solely an individual’s responsibility. For example, Helen, an 18-year-old Southeast Asian woman, identified the government as a purveyor of information and a motivating force for individuals to make change:

*I feel like the rates of obesity and health issues has increased and it can create a burden on the health care system, and the government maybe wants to prevent it. The government is saying let’s implement these strategies and kind of inform Canadian citizens how to prevent.*

On the other hand, some participants, like Cassie, perceived the government’s actions as potentially impacting the individual’s wellbeing, and perhaps even doing so intentionally to promote population health:

*I mean, we like to think that, like, the government wouldn’t try and make us feel bad about ourselves, but they definitely do because they don’t … want to be known for having an obese population. Like, that just doesn’t reflect well on our country as a whole. So maybe like a little bit of guilt isn’t so bad for, like, the overall health of an individual.*

The nine participants who highlighted the role diet-related initiatives play in preventing or reducing obesity seemed to cast higher weights and chronic disease as the outcomes of individual choice, with no differences by gender, body esteem, or disordered eating risk compared to the four participants who did not speak about obesity or chronic disease prevention as a key outcome of labelling policies.

### Role of the relationship with food in experiences with labelling

Participants described their *personal connections with food*; five participants detailed positive relationships with food (two had above-average disordered eating scores and ‘overweight’ perceptions), two described a neutral relationship with food (neither had elevated disordered eating or body esteem risks), and six described a relationship with food that was partly contentious (two had above-average disordered eating scores and one had history of an eating disorder). A partially contentious relationship with food may encompass positive elements, but included participants’ struggles with food in their daily lives, as Cynthia detailed: *‘I think I have a very strong relationship with food. Positive in that I eat, which is good. But negative in that I overeat, because I know I crave food a lot. I don’t necessarily always have the willpower.*’ All described the role of *food in their relationships with others* and the *societal pressures surrounding food* that occurred in public and/or private spaces, and identified pressures that subsequently interacted with their experiences with labels. Monica described: *‘I remember one time I was ordering UberEATS and I wasn’t just looking at the calorie labels, but I was conscious of what my friends were ordering as well, because they were getting smaller items.’* She then shared that she ordered a lower calorie item in line with her friends’ choices.

All participants also described *short- and long-term influences of labels* on their purchasing decisions and interaction with their relationships with food. Short-term influences affected the food purchase and thoughts or emotions during consumption. Long-term influences persisted after the meal was consumed (i.e., later that day and beyond). A hypothetical scenario was presented that asked participants to consider how a green, amber, or red label on their favourite food would affect their purchasing decision and associated feelings. Five participants reported the colour of the label would not affect their decision and eight said the red label would influence them to not purchase a food or consume a lesser amount of it. Eight participants indicated they had noticed nutrition information the last time they visited a restaurant and half of those stated it influenced what they ordered. Participants’ descriptions of seeing and choosing a low-calorie item left them with a fleeting positive emotion.

Negative long-term influences of labels were reported by six participants, including three with above-average disordered eating scores and four who identified with one or more of eating binges, self-induced vomiting, or excessive exercise to control their weight in the past 6 months. For some participants, like Maya, who both had an above-average disordered eating score and endorsed excessive exericse, the long-term consequences of labels influenced her eating later in the day:

*I wanted to take a dessert and, uh, it was a chocolate brownie, and it was like, a lot of calories I remember at the time … if the calorie thing wouldn’t have been there, I would have just taken it and not given a damn. But I did … It lingered till I was in the bus, and I was telling my friends: I should have taken that, I should have taken that, I should have taken that. … when I reached home, I had the craving again … So I just made my own custard and ate it, because I was craving something sweet.*

All participants identified at least one *negative impact of labels on their and/or others’ relationships with food*. Nine participants identified negative consequences only for others’ relationships with food (two had above-average disordered eating scores), while four reported that negative consequences may impact their own relationship with food (three had above-average disordered eating scores and/or a history with an eating disorder). Five participants explicitly stated labels may have adverse effects for individuals with eating disorders or contribute to an increase in eating disorders. Other identified consequences included driving more people to dieting, eliciting shame or embarrassment around eating, pressuring people to eat less, affecting how people think about food, targeting insecure populations (e.g., adolescent girls, people with higher weights), and leading people to fixate on calories rather than overall nutrition.

### Disordered eating and dieting as a lens in experiences with interventions

For several participants, their own disordered eating and/or weight management efforts interacted with their experiences with labelling interventions. Eight of the thirteen participants detailed instances of *overindulging and/or ‘bingeing*’ or having what they perceived as ‘too much’ of certain foods, which were usually low in nutritional value. Nine participants described instances of feeling *ashamed about and/or regret* related to their food choices. For some participants, this had to do with the shame of choosing a high-calorie food after seeing the label, but for Cynthia, it was related to the fact she consumed more than she wanted in an instance in which she did not have access to labels. Two other participants also hypothesized that the guilt around consuming certain foods might be alleviated if they had more knowledge and information to fight 'overconsumption,' because they would not have chosen that food.

Nine participants described behaviours or thoughts that aligned with attempts to modify their weight. The participants who reported *restrictive food and/or dieting behaviours* had varying scores on BESAA and EAT-26, but the participants whose eating pathology seemed most severe had higher EAT-26 scores and detailed how their dieting attempts intersected with their label-related experiences. Arjun, a 19-year-old South Asian man who perceives himself as underweight, detailed alternating cycles of a high-calorie diet to gain weight (‘bulking’) and severe caloric restriction and excessive exercise to ‘carve’ muscle out of the fat (‘cutting’). Speaking to the role of labels, Arjun described: *‘They help me choose high—higher calorie, low sugar, low cholesterol foods, uh, which is good.’* Similarly, other participants detailed how labels allowed them to achieve weight-related goals.

Twelve participants appeared to distance themselves from the influences of labels by *speaking for a friend*, describing one or more friends attempting to modify their eating patterns in ways that might influence their experiences with labels. Notably, six participants detailed that some young people are hyper-aware and conscious of their diets, and some have ‘unhealthy’ dietary patterns. However, access to labels was not necessarily seen as a route to healthy eating. For example, Jen detailed how her roommate may claim to look at labels when purchasing foods, but indicated she incorrectly interprets the nutrition information and proceeds to have an ‘unhealthy’ diet.

## Discussion

The findings of this study highlight the complexity underlying young adults’ interactions with calorie labels, with potential implications for the implementation of labelling policies. Participants exhibited both support for and skepticism of labels and identified ways in which they might help them or others make healthy choices or choices consistent with weight-related motivations. Participants who were women, had low body esteem, and/or had an elevated risk of disordered eating experienced labels and their after-effects differently than others. Such effects may have included prolonged negative feelings after encountering labels and greater pressure to alter their food purchasing and choices in the presence of others.

Consistent with participants’ support for labelling interventions, previous research demonstrates that young adults support food-related policies, such as calorie menu labels and informational campaigns [28, 43]. Moreover, research among university-educated young adults also demonstrates higher health literacy compared to the general population, which is in turn associated with higher use of nutrition and menu labels [44]. Nearly all participants in this study, however, expressed skepticism regarding the effectiveness of labels among their age group and/or the trustworthiness of the information. Several highlighted structural challenges to engaging in healthy eating (e.g., high food costs, limited food education in schools). Thus, perhaps their skepticism was rooted in the understanding that individualistic policies such as labelling cannot support healthy eating patterns without addressing structural barriers. At the same time, participants aligned with cultural narratives that assign responsibility for unhealthy eating to individual choice, underscoring tensions in experiences of seemingly straightforward interventions, such as calorie labels.

Participants identified potential negative consequences of labelling on their own and others’ relationships with food, mirroring a previous mixed-methods study exploring traffic light labelling among university-aged students [27]. Although 60% of participants supported labels and their implementation, nearly half expressed concern they may exacerbate eating disorders [27]. A study conducted at the same institution as the current study did not find that a brief labelling intervention exacerbated eating pathology [26]. However, based on our findings, the negative implications of labels may be more complex than eating pathology itself, and may include constructs such as one’s relationship with food, shame and embarrassment around eating with others, and fixating on calories versus overall nutrition. These potential outcomes are difficult to operationalize and assess, especially in short-term studies.

Given that eating disorders and disordered eating behaviours are highly prevalent globally [45], the findings of this study have implications for the implementation of calorie menu labelling policies, especially as several nations (such as the UK) prepare to implement mandatory calorie labelling in the near future [46]. The results echo other calls for complementary policies that target structural determinants [47], such as subsidies for healthful foods or restricting harmful food marketing practices. In considering unintended consequences, it is important to bear in mind that labels and similar interventions are implemented within a broader culture of healthism that reinforces individual responsibility for health, weight, and the moral value we ascribe to them [48, 49]. Consequently, it is necessary to address ‘diet culture’ in which calorie labelling and other interventions related to body weights are embedded. Otherwise, societal pressures that contribute to disordered eating may be perpetuated by such interventions and promote shame in public settings. Future research should explore the effects of labels on food and beverage purchasing and consumption decisions when an individual is alone versus in a group setting.

Although we attempted to employ maximum variation techniques through purposive sampling, the results are skewed to women (as is common in nutrition research). Selective bias in university-based study samples is not unique to this study, but limits generalizability to young adults more broadly. Recruitment was also limited to a narrow time period, and data collection and analysis may have been strengthened by a larger sample size. The study sample also consisted of a mix of domestic and international students at a single university, and does not represent the views of young adults in a North American context. However, these results provide context to guide future inquiry into the unintended consequences of weight-related interventions on a larger, more generalizable scale, and inform future food-related policies.

## Conclusion

This study suggests that young adults’ interactions with calorie labelling are complex, with some concern regarding their relationships with food and disordered eating. The findings contribute to the nascent literature on preventing potential unintended consequences related to eating disorders and negative psychosocial outcomes more broadly. Future investigations into the effectiveness of menu labelling should explore the roles of disordered eating, body esteem, and one’s relationship with food pre- and post-intervention and over extended periods of time, especially in the context of public health environments that reinforce healthism and focus on individual determinants of health.

## Supplementary Information

**Additional file 1.** Interview guide.

## Data Availability

The datasets generated and/or analysed during the current study are not publicly available due to the small sample size of the study and the identification of the institution from where data were collected, but deidentified data are available from the corresponding author on reasonable request.

## Abbreviations

BESAA: Body Esteem Scale for Adolescents and Adults (BESAA)
EAT-26: 26-item Eating Attitudes Test

## References

[1] GBD 2015 obesity collaborators (2017). Health effects of overweight and obesity in 195 countries over 25 years. N Engl J Med.

[2] Bombak AE (2014). The “obesity epidemic”: evolving science, unchanging etiology. Sociol Compass.

[3] Ramos Salas X, Forhan M, Caulfield T, Sharma AM, Raine K (2017). A critical analysis of obesity prevention policies and strategies. Can J Public Health.

[4] Crawford R (1980). Healthism and the medicalization of everyday life. Int J Health Serv.

[5] Medvedyuk S, Ali A, Raphael D. Ideology, obesity and the social determinants of health: a critical analysis of the obesity and health relationship. Crit Public Health. 2018;28(5):573–85.

[6] Talbot CV, Branley-Bell D. #BetterHealth: a qualitative analysis of reactions to the UK government’s better health campaign. J Health Psychol. 2022;27(5):1252–8. 10.1177/1359105320985576 PMC8978455 33426935

[7] Monaghan LF, Bombak AE, Rich E (2018). Obesity, neoliberalism and epidemic psychology: critical commentary and alternative approaches to public health. Crit Public Health.

[8] Raine KD, Ferdinands AR, Atkey K, Hobin E, Jeffery B, Nykiforuk CIJ (2017). Policy recommendations for front-of-package, shelf, and menu labelling in Canada: moving towards consensus. Can J Public Health..

[9] Jeong JY, Ham S (2018). Application of the health belief model to customers’ use of menu labels in restaurants. Appetite..

[10] McGeown L (2019). The calorie counter-intuitive effect of restaurant menu calorie labelling. Can J Public Health..

[11] Neumark-Sztainer D, Wall M, Larson NI, Eisenberg ME, Loth K (2011). Dieting and disordered eating behaviors from adolescence to young adulthood: findings from a 10-year longitudinal study. J Am Diet Assoc.

[12] Liechty JM, Lee MJ (2013). Longitudinal predictors of dieting and disordered eating among young adults in the U.S. Int J Eat Disord.

[13] Slof-Op ‘t Landt MCT, van Furth EF, van Beijsterveldt CEM, Bartels M, Willemsen G, de Geus EJ (2017). Prevalence of dieting and fear of weight gain across ages: a community sample from adolescents to the elderly. Int J Public Health.

[14] Nagata JM, Garber AK, Tabler JL, Murray SB, Bibbins-Domingo K (2018). Differential risk factors for unhealthy weight control behaviors by sex and weight status among U.S. adolescents. J Adolesc Health.

[15] Striegel-Moore RH, Rosselli F, Perrin N, DeBar L, Wilson GT, May A (2009). Gender difference in the prevalence of eating disorder symptoms. Int J Eat Disord.

[16] Raffoul A, Hammond D (2018). Correlates of weight-loss methods among young adults in Canada. Obesity..

[17] Lucan SC, DiNicolantonio JJ (2015). How calorie-focused thinking about obesity and related diseases may mislead and harm public health. An alternative. Public Health Nutr.

[18] Rubin R (2018). Will posting nutritional information on menus prod diners to make healthier choices?. JAMA..

[19] Moubarac JC, Batal M, Louzada ML, Martinez Steele E, Monteiro CA (2017). Consumption of ultra-processed foods predicts diet quality in Canada. Appetite..

[20] Romano KA, Swanbrow Becker MA, Colgary CD, Magnuson A (2018). Helpful or harmful? The comparative value of self-weighing and calorie counting versus intuitive eating on the eating disorder symptomology of college students. Eat Weight Disord.

[21] Courtney AL, PeConga EK, Wagner DD, Rapuano KM (2018). Calorie information and dieting status modulate reward and control activation during the evaluation of food images. PLoS One.

[22] Fawkes K, Levy J, Terry K, Edelstein S (2010). Female college students’ attitudes about body image and food labels and how they affect purchasing behavior. Top Clin Nutr.

[23] Christoph MJ, Loth KA, Eisenberg ME, Haynos AF, Larson N, Neumark-Sztainer D (2018). Nutrition facts use in relation to eating behaviors and healthy and unhealthy weight control behaviors. J Nutr Educ Behav.

[24] Larson N, Haynos AF, Roberto CA, Loth KA, Neumark-Sztainer D (2018). Calorie labels on the restaurant menu: is the use of weight-control behaviors related to ordering decisions?. J Acad Nutr Diet.

[25] Haynos AF, Roberto CA (2017). The effects of restaurant menu calorie labeling on hypothetical meal choices of females with disordered eating. Int J Eat Disord.

[26] Lillico HG, Hanning R, Findlay S, Hammond D (2015). The effects of calorie labels on those at high-risk of eating pathologies: a pre-post intervention study in a university cafeteria. Public Health.

[27] Seward MW, Block JP, Chatterjee A (2018). Student experiences with traffic-light labels at college cafeterias: a mixed methods study. Obes Sci Pract.

[28] Bhawra J, Reid JL, White CM, Vanderlee L, Raine K, Hammond D (2018). Are young Canadians supportive of proposed nutrition policies and regulations? An overview of policy support and the impact of socio-demographic factors on public opinion. Can J Public Health.

[29] Acton RB, Hammond D (2018). Do consumers think front-of-package “high in” warnings are harsh or reduce their control? A test of food industry concerns. Obesity..

[30] Ebneter DS, Latner JD, O’Brien KS (2011). Just world beliefs, causal beliefs, and acquaintance: associations with stigma toward eating disorders and obesity. Personal Individ Differ.

[31] Seward MW, Soled DR. Unintended consequences in traffic-light food labeling: a call for mixed methods in public health research. J Am Coll Heal. 2020;68(5):465–7. 10.1080/07448481.2019.1583238 30908133

[32] Creswell JW (2014). Designing research: mixed methods procedures. Research design: qualitative, quantitative, and mixed methods approaches.

[33] Malam S, Clegg S, Kirwan S, McGinigal S (2009). Comprehension and use of UK nutrition signpost labelling schemes.

[34] Ontario Ministry of Health. Menu Labelling Protocol, 2020. Toronto: Ontario Ministry of Health; 2020.

[35] Lee KM (2021). Supporting healthy and sustainable campuses: examining food and nutrition interventions in real-world settings.

[36] Palinkas LA, Horwitz SM, Green CA, Wisdom JP, Duan N, Hoagwood K (2015). Purposeful sampling for qualitative data collection and analysis in mixed method implementation research. Adm Policy Ment Health Ment Health Serv Res.

[37] Hill LS, Reid F, Morgan JF, Lacey JH (2010). SCOFF, the development of an eating disorder screening questionnaire. Int J Eat Disord.

[38] Canadian Institutes of Health Research (2015). Sex, Gender and Health Research Guide: A Tool for CIHR Applicants.

[39] Statistics Canada (2013). Canadian Community Health Survey - Annual Component (CCHS).

[40] Mendelson BK, Mendelson MJ, White DR (2001). Body-esteem scale for adolescents and adults. J Pers Assess.

[41] Berland NW, Thompson JK, Linton PH (1986). Correlation between the EAT-26 and the EAT-40, the eating disorders inventory, and the restrained eating inventory. Int J Eat Disord.

[42] Braun V, Clarke V (2006). Using thematic analysis in psychology. Qual Res Psychol.

[43] Diepeveen S, Ling T, Suhrcke M, Roland M, Marteau TM (2013). Public acceptability of government intervention to change health-related behaviours: a systematic review and narrative synthesis. BMC Public Health.

[44] Cha ES, Kim KH, Lerner HM, Dawkins CR, Bello MK, Umpierrez G (2014). Health literacy, self-efficacy, food label use, and diet in young adults. Am J Health Behav.

[45] Galmiche M, Déchelotte P, Lambert G, Tavolacci MP (2019). Prevalence of eating disorders over the 2000–2018 period: a systematic literature review. Am J Clin Nutr.

[46] Robinson E, Marty L, Jones A, White M, Smith R, Adams J (2021). Will calorie labels for food and drink served outside the home improve public health?. BMJ..

[47] Gore D, Kothari A (2012). Social determinants of health in Canada: are healthy living initiatives there yet? A policy analysis. Int J Equity Health.

[48] LeBesco K (2011). Neoliberalism, public health, and the moral perils of fatness. Crit Public Health.

[49] Monaghan LF. Re-framing weight-related stigma: from spoiled identity to macro-social structures. Soc Theory Health. 2016;15(2):182–205.

