# Supplementary material for: “Maybe a little bit of guilt isn’t so bad for the overall health of an individual”: a mixed-methods exploration of young adults’ experiences with calorie labelling
Source: BMC Public Health. 2022 May 10;22:938. doi: 10.1186/s12889-022-13364-w (PMC9092873; doi:10.1186/s12889-022-13364-w)
Supplement: Supplementary file 1 — Additional file 1. Interview guide. [file 12889_2022_13364_MOESM1_ESM.docx]

***Interview guide***

|  | **Question type** | **Question/prompt** | **Probes** |
| --- | --- | --- | --- |
| 1. | Warm-up | What is your favourite food to cook or eat? |  |
| 2. | Transition | How often do you cook your own food at home? | - What kinds of food do you cook? - Tell me more about the preparation of these foods, like how you make them or what kinds of ingredients you use. |
| 3. | Main question | Some people describe having a “relationship with food”, which captures how they interact with food in their daily lives and the meaning, emotions, and value that they get from food.  In your own words, how would you describe your relationship with food? | - What feelings do you associate with food? (i.e., eating, preparing, buying) - Can you tell me more about that? |
| 4. | Follow-up | Are there any foods that make you feel good or bad? | - What are they? - Do these foods make you feel the same way in all situations? Eating alone versus with friends, eating at home versus in a restaurant, etc. How/how not? - Do these foods influence your interactions with other people? Friends, family, roommates, etc. How/how not? |
| 5. | Transition | What do you think about Canadians’ diets, or how Canadians overall relate to food? | - Can you tell me more about that? - Do you think they are healthy? Why/why not? |
| 6. | Main question | Recently, the Government of Canada began working on a *Healthy Eating Strategy* to improve the overall diets of Canadians. Have you heard of this initiative? | - (If yes) Could you tell me what you know about this initiative? Or what it entails? - (If no or unsure) It’s a national framework for policies and recommendations to encourage ‘healthy eating’. Some examples of policies and recommendations include dietary guidance (like a recent update to Canada’s Food Guide), calorie labelling, and changing nutrition labels on packaged foods. |
| 7 | Follow-up | Why do you think governments develop interventions or make policies related to food? | - What impact do you think these policies have? |
| 8. | Follow-up | How do these food-related policies affect you? | - Do you think they influence your food-related behaviour? Why/how? - Think back to our discussion about your “relationship with food”. Can you tell me more about the role that policies might have on your relationship with food? |
| 9. | Main question | You recently participated in a study in a residence cafeteria on campus. Can you tell me more about that? | - (If yes) Did you notice any changes in the cafeteria around the time of the study? - (If no) In that study, labels within the cafeteria you visited may have been modified to include nutrition information for each food and beverage. These labels indicated the number of calories in each item, possibly using red, amber/yellow, and green to identify high, medium, and low-calorie items. Do you remember this now? - What do you think the purpose of that study was? |
| 10. | Follow-up | I would like you to close your eyes and think back to that period of time when the labels in the residence cafeteria might have been different than usual. You are approaching the place where you choose the foods and beverages you are going to purchase. You see that the cafeteria is offering [*participant’s favourite food to cook from Q1*]. The label for the item shows its calorie content with a green circle. Please open your eyes. Can you tell me how you would feel in this situation? | - Can you tell me the first thought that popped into your head? Why do you think this was first? How did that make you feel? - Do you think this feeling would linger, or go away quickly? Why/why not? - Would you still choose this food? Why/why not? - What if the circle was red or amber? How would you feel? Would you still choose this food? Why/why not? |
| 11. | Main question | Aside from the recent experiment in your residence cafeteria, recent laws have made it so that restaurants with more than 20 locations must display calories for each menu item. Have you noticed this in any food settings you’ve visited or on sites or apps you’ve used to order food? Can you name a specific time when you saw calorie labels and tell me how you felt or reacted? | - How did it make you feel? Did this reaction influence your purchasing decision? (i.e., did you order something different than you might have otherwise?) - Did this feeling linger, or did it go away quickly? |
| 12. | Follow-up | You mentioned that this type of scenario, where you encounter a calorie label in a restaurant or on an app, would make you feel [*name participant’s feeling*]. Again, I would like you to think about your own relationship with food overall, which you mentioned is [*summarize response to Q3*]. Can you tell me about how seeing labels with calorie content makes you feel, considering your relationship with food? | - What do you think the aim of this intervention (calorie labels) is? - How do you think calorie labelling might affect other people’s relationship with food? Are there any positive/negative implications? |
| 13. | Wrap-up | Is there anything else you would like to talk about? |  |
